# An Intervention on Anxiety Symptoms in Moderate Alzheimer’s Disease through Virtual Reality: A Feasibility Study and Lessons Learned

**DOI:** 10.3390/ijerph20032727

**Published:** 2023-02-03

**Authors:** Desirée Sánchez-Nieto, Sergio Castaño-Castaño, Remedios Navarro-Martos, Esteban Obrero-Gaitán, Irene Cortés-Pérez, Francisco Nieto-Escamez

**Affiliations:** 1 VIVALDI Day Stay Unit, Avda. Reino de España 184, Roquetas de Mar, 04740 Almería, Spain; 2 Department Psychology, University of Oviedo, Plaza Feijoo S/N, 33003 Asturias, Spain; 3 Department Psychology, University of Almería, Carretera del Sacramento S/N, 04120 Almería, Spain; 4 Department Health Sciences, University of Jaén, Campus Las Lagunillas S/N, 23071 Jaén, Spain; 5 Center for Neuropsychological Assessment and Rehabilitation (CERNEP), University of Almería, Carretera del Sacramento S/N, 04120 Almería, Spain

**Keywords:** dementia, Alzheimer’s disease, anxiety, non-pharmacological intervention, virtual reality

## Abstract

(1) Background: Although cognitive impairment is considered the core deficit of dementia, anxiety disorders also have a negative influence on the social and daily life of the affected population. We have explored the exposure of relaxing scenarios in immersive Virtual Reality (iVR) as an intervention strategy for people with moderate Alzheimer’s disease. (2) Methods: Three participants were recruited from a day center to participate in a five-week study, which included a Pre- and Post-evaluation with the Montreal Cognitive Assessment (MoCA), Neuropsychiatric Inventory-Questionnaire (NPI-Q), Clinical Dementia Rating Scale (CDR), Global Deterioration Scale (GDS), Hamilton Anxiety Rating Scale (HARS), State-Trait Anxiety Inventory (STAI), and the anxiety subdomain of the Neuropsychiatric Inventory (NPI). Participants’ heart rate, oxygen saturation, arterial pressure, and respiratory rate were also monitored during intervention sessions. Three virtual scenarios from Nature Treks VR were used as the intervention over three weeks (a total of nine sessions). (3) Results: Post-intervention anxiety assessment showed a light reduction in psychological anxiety in the HARS questionnaire. A light reduction in heart rate was also observed during the exposure to iVR. (4) Discussion: The use of virtual scenarios was a satisfactory experience for all the participants. Preliminary data point to a relaxing effect of iVR scenarios and a potential reduction in psychological anxiety, but further research is required to confirm the efficacy of the intervention.

## 1. Introduction

Dementia is a chronic disease that consists of a persistent deterioration of higher brain functions such as memory, language, and decision-making [1]. It is an acquired disorder that evolves gradually, being more common in older ages [2]. Dementia reduces the autonomy of the patient, who becomes increasingly dependent on others for even the smallest daily activity, and places a high burden on caregivers [3]. According to the World Health Organization (WHO), dementia is the leading cause of disability and dependency among the elderly worldwide [4].

Data from the WHO indicate that dementia affects 55 million people worldwide. Each year approximately 10 million new cases are registered, and it is forecast that there will be 78 million cases in 2030 and 139 million in 2050, with Alzheimer’s disease (AD) being the most common form of dementia, with 60–70% of cases [4]. Although AD is primarily a neurocognitive disorder, it also entails different neuropsychiatric symptoms (NPS), including apathy, depression, anxiety, agitation, aggression, and psychosis [5]. Much research has focused on symptoms such as depression [6,7], apathy [8,9], or agitation [10,11], but the relation between anxiety and AD has received much less attention.

A meta-analysis study reported a pooled prevalence estimate for anxiety in AD of 39% [12], with generalized anxiety (GAD) as the usual type. The severity of anxiety seems to be stable or increased during the course of dementia. At a severe stage of dementia, anxiety gradually decreases [13,14,15]. GAD is characterized by persistent and excessive worry about a number of different things. Individuals with GAD find it difficult to control their worry and have at least three of the following associated anxiety symptoms for 6 months: restlessness, fatigue, concentration problems, irritability, muscle tension, and sleep disturbance [14]. Mild-to-moderate AD patients with GAD show significantly higher scores of depression, irritability, overt aggression, mania, and pathological crying than AD patients without GAD but with a similar age and cognitive deterioration, being restlessness, muscle tension, fears, insomnia, and physical complaints the most prominent symptoms [16,17]. However, Calleo et al. [18] have suggested that only muscle tension and fatigue are specific for GAD in patients with dementia.

Both psychological and biological mechanisms have been described as probable causes of anxiety in patients with dementia [19]. It has been reported that a reduction in their coping ability and loss of control in daily living [20], which may be linked to the atrophy of the limbic structures produced by high levels of cortisol due to stress [21]. Thus, patients with dementia have less control over their feelings and how to express them. They have difficulties managing grief, overreact to things, show sudden mood changes, or feel irritable, which is accompanied by dysfunctional coping strategies such as wandering, withdrawal, avoidance, and aggressive actions [22].

Currently, there is no evidence about the efficacy of pharmacological treatment of anxiety in patients with dementia. Non-pharmacological approaches such as cognitive behavior therapy have been proposed as an alternative for elderly patients with anxiety comorbid with mild dementia or immediately after the diagnosis of dementia [19]. More recently, Dimitriou et al. [23] have shown that music therapy, exercise, aromatherapy, and massage were effective, in that order, as a treatment to reduce anxiety in patients with dementia. Another promising tool that can be included among non-pharmacological strategies is virtual reality. Immersive virtual reality (iVR) enables people to escape their restricted physical realities and be transported to calming places. Thus, iVR has been used to reduce anxiety in different populations and pathologies. Chirico et al. [24] reported that exposure to nature images in iVR was more effective in reducing anxiety in breast cancer patients than music therapy and nonintervention. Appel et al. [25] explored the effect of exposing older adults to nature scenes through an iVR headset. The authors reported that participants experienced an increase in positive emotions, feeling relaxed and content. Niki et al. [26] reported that reminiscence therapy through iVR reduced anxiety in elders. Nevertheless, in this study, participants’ cognitive function was mostly preserved. More recently, Brimelow et al. [27] found that iVR induced a positive emotional response in nursing home residents. Therefore, the goal of the present study is to assess the efficacy of iVR (nature scenes) in reducing the anxiety of patients diagnosed with moderate dementia.

## 2. Materials and Methods

### 2.1. Participants

Three participants of Spanish nationality were selected from VIVALDI Day Stay Unit (Roquetas de Mar, Spain). All the participants had been diagnosed with AD and scored between five and six on the Global Deterioration Scale. The participants were females aged between 64 and 88 years. The presence of any of the following symptoms or diagnostics was considered an exclusion criteria: presence of seizures, ideational, ideomotor or oculomotor apraxia, clinical depression, hallucinations, high aggressiveness, injuries in shoulders, neck, or head, and previous experience using iVR devices. All the participants and their legally responsible relatives were informed about the research characteristics and gave their informed consent before participation. None of the participants received monetary compensation. See Table 1.

### 2.2. Instruments

#### 2.2.1. Neuropsychological, Neuropsychiatric, and Attitude Questionnaires

The Spanish version of the Montreal Cognitive Assessment test (MoCA) [28,29], Neuropsychiatric Inventory-Questionnaire (NPI-Q) [30,31], Clinical Dementia Rating Scale (CDR) [32,33], Global Deterioration Scale [34,35], Hamilton Anxiety Rating Scale (HARS) [36,37], the short version of the State-Trait Anxiety Inventory (STAIr) developed by Fernández-Blázquez et al. [38], and the Neuropsychiatric Inventory (NPI) [39,40], were used to assess participants’ cognition and psychopathology (see Table 2).

The MoCA test assesses the following cognitive domains: naming, episodic memory, language, attention, abstract thinking, visuospatial and executive function, and orientation. Nasreddine et al. [29] established a cut-off point to differentiate cognitive damage from normal aging with a score of 26. 

The NPI-Q is a validated clinical instrument for the evaluation of neuropsychiatric symptoms in dementia. 

The CDR assesses 6 domains: memory, orientation, problem-solving, functioning at home/hobbies, personal care, and community relations. The evaluation of these areas yields a classification into 5 different stages, with a score of 0 indicating the absence of dementia and a score of 3 indicating the presence of severe dementia. 

The GDS classifies the patients into 1 of 7 possible levels, where 1 to 3 indicate pre-dementia phases and levels 4–7 indicate dementia phases. 

The HARS is one of the most widely used psychological assessment instruments in research and clinical practice. It allows us to differentiate between psychological anxiety and somatic anxiety. 

The STAIr is a reduced version of the STAI. It is based on a theoretical model that recognizes two components of anxiety: state anxiety as a temporary emotional state (corresponds to STAI items 27, 29, 30, 31, 32, 33, and 38) and trait anxiety as a constant personal tendency (corresponding to STAI items 1, 5, 8, 12, 18, and 19). 

For the NPI, only items related to the “anxiety” sub-domain were administered to participants’ caregivers. 

An ad-hoc questionnaire was developed and used at the end of the study to assess participants’ acceptance of the activity. The questionnaire included three closed questions: “Did you feel well while you were watching the landscapes in VR?”, “Did you feel bad while you were watching the landscapes in VR?”, “Would you like to continue using VR?”. The participants were given three possible responses: “Nothing”, “Something”, “Much”. A second questionnaire was developed to request the opinion of the neuropsychologist at the center. This questionnaire included three closed questions: “Have the patients expressed pleasure with the VR-based intervention?”, “Have the patients expressed displeasure with the VR-based intervention?”, “Do you think it would be positive to continue using the VR-based intervention?”, with three possible answers for each question: “Nothing”, “Something”, “Much”. There were also three open questions: “Which benefits do you perceive in this activity?”, “Which problems do you perceive in this activity?”, “Would you make any change in the intervention?”. 

#### 2.2.2. Hardware and Software

A set of three wristbands (Color Health, Leotec, Barcelona, Spain) were used to register participant’s physiological responses: heart rate, blood oxygen saturation (SpO2), blood pressure (diastolic and systolic), and breathing rate (see Table 2). The smart bands were linked through Bluetooth to three smartphones running the software FitTCloud (version 1.7.3.). 

The software Nature Treks VR v. 1.29 (Greener Games, Telford, UK) is a commercially available application consisting of a suite of color-themed natural environments. Nature Treks aims to promote relaxation through an immersive experience while the user is within a natural environment. The selected environments were Red Fall, Green Meadows, and Blue Ocean. 

A set of three Oculus Go 64GB headsets (Oculus VR, Menlo Park, CA, USA) were used to display the virtual environments. These headsets are equipped with an LCD (2560 × 1440 pixels), a Qualcomm Snapdragon 821 microprocessor, an Adreno 530 graphic card, and a refresh frequency of 72 Hz. 

### 2.3. Procedure

**Figure 1:**
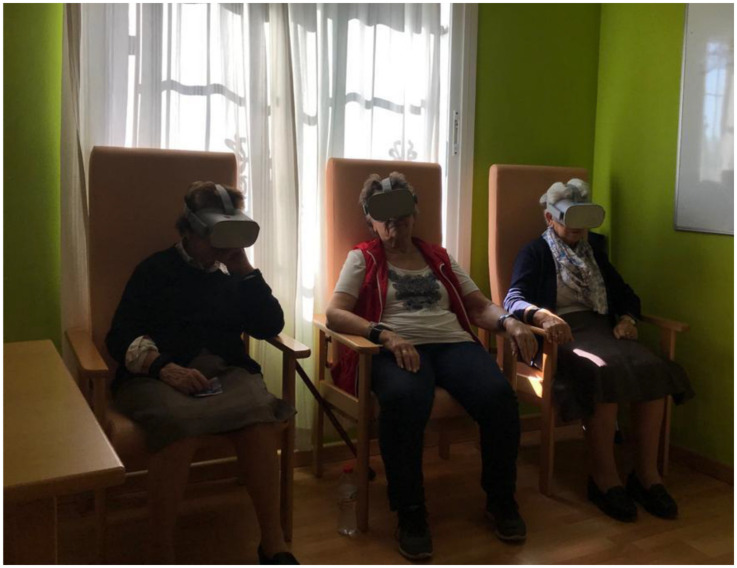
Participants watching the virtual scenes during the intervention.

The study lasted five weeks (see Table 3) and was carried out in the day center that the participants attended daily. It consisted of three phases. (1) Neuropsychological (MoCA, NPI-Q, CDR, GDS) and anxiety (HARS, STAIr, NPI) baseline assessment, which took one week (Monday, Wednesday, and Friday). (2) Subsequently, the intervention phase was carried out for three weeks, three days per week. In total, nine iVR sessions were carried out. Three different scenarios from Nature Treks were employed: Red Fall, Green Meadow, and Blue Ocean. For each session, participants were moved to a quiet room in dim light and were sat on a comfortable chair, always around 11 AM (see Figure 1). All three scenarios were employed every week (a different one was displayed in each session). In every session, participants were exposed to the virtual scenario for 15 min, and all the physiological measures were registered 5 times. Once, after they arrived at the room and sat in the chair before they wore the headset (pre), then, while they were watching the virtual scenes at three different times (5, 10, and 15 min after they started watching the scenes), and finally, 5 min after they had finished watching the scenes and the headset had been removed (post). Then, the STAIr state items of the questionnaire were assessed. (3) The study ended with the post-assessment phase of neuropsychological and anxiety variables, one week after the intervention phase. The same protocol as in the baseline assessment was followed (with the exception of the STAIr state). 

During every intervention session, once all the participants were seated on their chairs and wore their wristbands, the researcher switched the Oculus Go headset on, put it on, and ran the iVR application, making sure the headset and the software were working properly. Then, the researcher took off the headset and put it on the participants. The participant was asked what she was watching through the headset and if the images looked good to ensure that the software continued running. This procedure was repeated for all three participants. 

A researcher different from the one responsible for the intervention was in charge of participants’ neuropsychological and anxiety evaluations in the pre- and post-intervention assessment phases. A comparison between pre- and post-intervention scores was performed for all the questionnaires. 

### 2.4. Analysis

Quantitative data were analyzed by using the software JASP 0.16.4.0. A non-parametric Wilcoxon signed-rank test was used to compare pre- and post-intervention scores obtained in neuropsychological and anxiety assessments according to the following hypotheses: MoCA (pre<post), NPI-Q (pre>post), CDR (pre>post), GDS (pre>post), HARS psychological (pre>post), HARS somatic (pre>post), STAIr trait (pre>post), NPI (pre>post). The non-parametric Friedman test for repeated measures was used to test if there were changes in physiological measures associated with the iVR experience (five times every session: pre, 5 min, 10 min, 15 min, and post) and if STAIr state baseline was different with regard to intervention sessions. If significant differences were obtained in the Friedman test, Connover’s Post Hoc comparisons were conducted. 

## 3. Results

Regarding participants’ behavior, there were no dropouts, and all the participants showed acceptance behaviors during the activity. All the participants made positive verbalizations and referred to positive feelings during and after completing the activity (e.g., “I have enjoyed watching the landscapes and the animals”). There were a few complaints about using the equipment (e.g., “the headset will mess up my hairstyle”). 

Regarding neuropsychological and psychiatric results, a Wilcoxon signed-ranks test showed no significant differences when the pre-intervention assessment and the post-intervention assessment scores were compared for neuropsychological and anxiety measures (see Table 4). A small but non-significant reduction in HARS psychological score was observed in the post-evaluation. 

**Figure 2:**
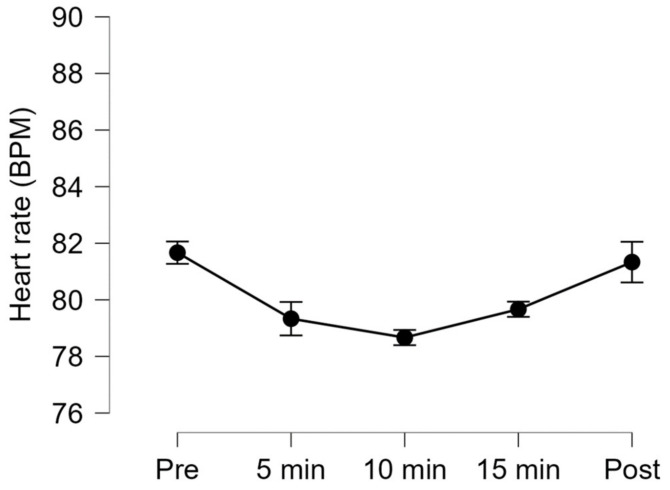
Heart rate values (Mean ± SEM) at different time points before, during, and following iVR exposure to relaxing scenarios.

Regarding physiological variables, Friedman’s test showed that recording time had a significant effect on participants’ heart rates. However, Connover’s post hoc pairwise comparisons showed no significant differences for any comparison (see Table 5). Figure 2 illustrates a light reduction of participants’ heart rates during the exposure to iVR. 

All the participants responded to the questionnaire developed to assess their acceptance of the iVR-based activity (see Table 6). All of them expressed that they had a positive feeling during the experience. Only one participant reported some discomfort wearing the headset for the required time. For this reason, this participant expressed less enthusiasm about continuing to use iVR in the future. All the participants said that they enjoyed watching beautiful landscapes without moving from the daycare center. All of them mentioned that the activity was novel and that they felt like they were in the country. 

The feedback reported by the neuropsychologist of the daycare center was positive regarding her perception of participants’ emotions, motivation, and the usefulness of iVR as a therapeutic tool (see Table 7).

## 4. Discussion

In the present study, we have tested the feasibility of exposing AD patients to iVR relaxing scenarios as a valid instrument for reducing anxiety levels. According to participants’ behavior and verbalizations during and after the activity, we can affirm that it was positively accepted by AD patients. All participants reported that they enjoyed the experience, as it was new for them, and they were allowed to watch beautiful scenes. This stresses the validity of iVR in removing physical barriers that impede elders from having access to new experiences. Moreover, none of the participants showed adverse side effects such as nausea, dizziness, disorientation, or confusion, which is in accordance with a previous work by Appel et al. [25], who tested tolerability, comfort, and ease of use of the HMD in older adults with variable levels of cognitive and physical impairment. 

As was expected, the exposure to nature-based iVR scenarios was attractive, maintaining participants’ motivation and adherence during the whole study. No participant showed reluctance to participate during the course of the research. Among the factors that would have influenced such good acceptance are the realism and quality of images and sounds. Moreover, during the exposure to the iVR scenarios, patients made positive verbalizations and described their experience, telling what they were watching. Sometimes they reminisced about other memories as a result of the iVR experience, particularly when animals were shown in the scene. Their interest in communicating their experience to others indicates that joint or multi-user experiences should be considered in future iVR interventions [25]. 

Previous studies have shown that exposure to iVR is effective in treating psychiatric disorders by inducing relaxation [41]. It is also known that the characteristics of the stimuli people are exposed to are of paramount importance in order to elicit positive emotions and foster psychological well-being. For instance, Ulrich et al. [42] described that natural scenes produce positive emotional states in contrast to urban scenes, which can be explained from an evolutionary perspective, stating that humans have an unlearned predisposition to natural content. A recent work by Dakoure et al. [43] used a virtual landscape as a relaxing treatment for people with subjective cognitive decline. In this work, the authors reported a reduction in participants’ mean frustration measured with Emotiv EEG during the exposure to the software Savannah VR.

Our work has been conducted as a small pilot study intended to assess the effectiveness of immersive natural scenarios in improving Alzheimer’s patients’ emotional status. Moreover, in contrast to previous studies [25,26,27], only patients suffering from moderate or moderately severe dementia were included in the current work. In our case, the comparison between the pre- and post-intervention of both neuropsychological and anxiety scales did not reveal significant differences. Nevertheless, a marginally significant reduction in the HARS psychological score was observed following the intervention. Our results indicate that the intervention was not effective in producing a significant improvement in cognitive variables or neuropsychiatric symptoms in the participants, although the HARS psychological scores point to a trend for a reduction of patients’ psychological anxiety. This result is not unexpected due to the level of the cognitive and behavioral decline of the participants. 

In contrast to data obtained from questionnaires, physiological measures offer the important advantage of continuous monitoring of responses during the intervention. The only physiological variable showing an observable change was the participants’ heart rates. A small decrease in mean heart rate was observed during the exposure to iVR scenarios in all participants. Although post-hoc analyses did not reach significance in pairwise comparisons, the observed heart rate reduction is in line with a recent study reporting a reduction of 1.2 bmp in cancer patients exposed to iVR intervention [44]. 

Regarding participants’ subjective perception of the intervention, they mostly reported an increase in wellness during the intervention. Only one of them expressed some annoyance, mainly due to the discomfort caused by the HMD. Similarly, the neuropsychologist responsible for the participants reported they had expressed pleasure regarding the intervention, and the activity could be useful in promoting emotional benefits for them. 

We must acknowledge some limitations in the present study. First, the small sample size impedes generalizing the present results. Secondly, the short duration of the intervention, being carried out in a time of only three weeks. Thirdly, the post-intervention assessment was carried out shortly after the treatment had been conducted. Fourthly, additional measures should be considered, both regarding neuropsychological and psychiatric instruments and physiological variables. This work was conceived as a pilot study aimed at assessing the feasibility of the procedure. ‘The observed encouraging results support further research with an RCT based on this training procedure’. This will increase the objectivity and generalizability of these preliminary results.

## 5. Conclusions

The present pilot study demonstrates the feasibility of our intervention procedure as a strategy to address anxiety symptoms in moderate cases of AD. Patients showed a willingness to perform the activity and judged it positively, as well as the neuropsychologist in charge of the patients. Moreover, despite the reduced number of participants and the short duration of the intervention, a marginally significant improvement in HARS psychological score was observed in the post-intervention assessment, and a reduction in patients’ heart rates was observed during the intervention.

## Figures and Tables

**Table 1:** Participants’ characteristics.

Participant	Sex	Age	Education	Medication
BA	Female	88	Primary (literate)	Memantine 20 mg
BE	Female	86	Primary (literate)	Memantine 20 mg
BF	Female	64	Primary (literate)	Memantine 20 mg

**Table 2:** Dimensions assessed and instruments used.

Psychological and Physiological Dimensions	Instrument
Cognitive functions Level of dementia Anxiety Psychopathology Heart rate, SpO2, blood pressure, breathing rate	MoCA CDR, GDS NPI, STAIr, HARS NPI-Q Wristband

**Table 3:** Phases of the study.

Pre-Assessment (Baseline) 1st Week	Intervention 2nd, 3rd, 4th Week	Post-Assessment 5th Week
MoCA NPI-Q CDR GDS HARS STAIr trait STAIr state NPI	Exposure to iVR (9 sessions) + Physiological recordings (pre, 5, 10, and 15 min, and post) + STAIr state	MoCA NPI-Q CDR GDS HARS STAIr trait NPI

**Table 4:** Neuropsychological and anxiety scales results.

Test	Timing	Mdn	Min	Max	W	*p* Value	Rank-Biserial Correlation
MoCA	Pre Post	3 3	3 3	7 6	1.000	0.977	1.000
NPI-Q	Pre Post	18 17	15 14	44 44	1.500	0.681	0.000
CDR	Pre Post	2 2	2 2	3 3	(a)	-	-
GDS	Pre Post	5 5	5 5	6 5	(b)	-	-
HARS psy	Pre Post	14 13	11 9	14 13	6.000	0.087	1.000
HARS som	Pre Post	6 4	0 0	6 6	1.000	0.500	1.000
NPI	Pre Post	0 0	0 0	1 1	(a)	-	-
STAIr trait	Pre Post	3 3	2 2	3 3	(a)	-	-
STAIr state	Pre Week 2 Week 3 Week 4	2 2 2 1	1 1 1 1	2 2 2 2	**Friedman Test** χ^2^ (3) = 1.286	*p* = 0.733	

Mdn = median; (a) The variance in Difference between Pre and Post is equal to 0; (b) The variance in Post is equal to 0; HARS psy = scores corresponding to psychological anxiety items 1,2,3,4,5,6,14; HARS som = scores corresponding to somatic anxiety items 7,8,9,10,11,12,13; NPI = scores corresponding to anxiety subdomain; STAIr trait subscale; STAIr state subscale.

**Table 5:** Physiological measures results.

Physiological Measure	Friedman Test	*p*-value	Connover’s Post Hoc
Heart rate	χ^2^(4) = 10.538	0.032 *	>0.05 for all pairwise comparisons (Holm correction)
Breathing rate	χ^2^(4) = 7.721	0.102	-
Diastolic blood presure	χ^2^(4) = 6.667	0.155	-
Systolic blood pressure	χ^2^(4) = 5.037	0.284	-
SpO2	χ^2^(4) = 3.000	0.558	-

* *p* < 0.05.

**Table 6:** Number of responses to each item of the questionnaire developed to register participants’ acceptance of iVR.

Item	“Nothing”	“Something”	“Much”
“Did you feel well while you were watching the landscapes in VR?”	0	0	3
“Did you feel bad while you were watching the landscapes in VR?”	2	1	0
“Would you like to continue using VR?”	0	1	2

**Table 7:** Neuropsychologists’ feedback about the iVR-based intervention.

Item	“Nothing”	“Something”	“Much”
“Have the patients expressed pleasure with the VR-based intervention?”			X
“Have the patients expressed displeasure with the VR-based intervention?”	X		
“Do you think it would be positive to continue using the VR-based intervention?”			X
“Which benefits do you perceive in this activity?”	Mainly relaxation and motivation for new activities.		
“Which problems do you perceive in this activity?”	None.		
“Would you make any change in the intervention?”	Increase the number of participants and the possibility of interactions.		

## Data Availability

The data that support the findings of this study are available from the corresponding author upon reasonable request.
